# Causes of delay in proper treatment of patients with undescended testis 

**Published:** 2011

**Authors:** Seyyed Mostafa Shiryazdi, Abbas Modir, Soheil Benrazavi, Nooshin Moosavi, Mohammad Kermani-Alghoraishi, Rahil Ghahramani

**Affiliations:** 1 Department of Surgery, Shahid Sadoughi University of Medical Sciences, Yazd, Iran.; 2 Student Research Committee, Shahid Sadoughi University of Medical Sciences, Yazd, Iran.

**Keywords:** *Delayed diagnosis*, *Undescended testis*, *Orchiopexy*, *Infant*

## Abstract

**Background:** Undescended testis (UDT) is the most common endocrine disorder in male children. Delayed diagnosis and treatment of UDT lead to complications such as infertility, malignancy and testis rotation.

**Objective:** The aim of this study was to evaluate the causes of delay in proper treatment of patients with undescended testis in our population.

**Materials and Methods:** An observational, descriptive, cross sectional study of 143 male patients, who applied to Shahid Sadoughi University Hospitals for orchiopexy operation was performed. The maximum recommended age for orchiopexy was 18 months.

**Results: **The mean age at referral was 5.34 years. Only 44 (30.8%) cases were operated on before the age of 18 months. The most common reasons of delay in treatment were absence of early diagnose (42.5%), parent's unawareness of surgery necessity and its complications associated (33.7%) and parent's disregard (23.5%). Only 19.6% of patients were diagnosed at born in the hospital. 49% of parents had the correct information for proper operation age and 40.6% of them had enough information about necessity of surgery and side effects of disease. Parent’s literacy, place of living and type of cryptorchidism had no significant relation with delay diagnosis (p> 0.05).

**Conclusion: **These results revealed that late diagnosis by physician and lack of insight of parents are the main reasons in delayed diagnosis and treatment of UDT. Therefore, education of parents and careful physical examination of the babies at birth and regular follow-up until 18 months can prevent the delay in diagnosis.

## Introduction

Cryptorchidism is the most common endocrine disorders in male pediatrics (1). Cryptorchidism literally means hidden or obscure testis and generally refers to an undescended or maldescended testis. Overall, 2% to 4% of term male newborns have cryptorchidism and it comes down to 1% until 6 months to 1 year approximately. It is uncommon to descending testes after the first year of life spontaneously (2). Predisposing factors for cryptorchidism including low birth weight, small for gestational age, preterm delivery and maternal exposure to estrogen during the pregnancy (3). A recent study found that almost 23% of index patients with undescended testes (UDT) had a positive family history of cryptorchidism (4). In cryptorchidism, the most useful determination is whether the testes are palpable upon physical examination. Approximately 80% of undescended testes are palpable and 20% are nonpalpable. Nonpalpable testes may be intra-abdominal or absent. Palpable testes may be undescended, ectopic, or retractile (5). Therefore a gentle physical examination and screening by physician is needed. Treatment includes medical therapy (Human chorionic gonadotropin and Gonadotropin-releasing hormone) and surgical therapy. Recently, any undescended testis after the age of 6 months should be referred for orchiopexy (6) because, cryptorchidism may have the long-term consequences on testicular function, including disturbed spermatogenesis and risk of testicular cancer, even after successful treatment (7). 

 Undescended testes are also more susceptible to testicular torsion and infarction and inguinal hernias (8). Currently, the fundamental problem in these patients is the delay in referring to treatment, what different factors are involving. Missed diagnoses at screening and delayed referral by physicians are the major factors for this delay. (9-14). The aim of this study was to evaluate the causes of delay in proper treatment of patients with UDT in our population.

## Materials and methods

A observational, descriptive, cross sectional study of 143 male patients, age between 20 days to 46 years who applied to Shahid Sadoughi University Hospitals (Yazd, Iran) for orchiopexy operation was performed. Diagnosis of UDT was based on physical examination; sonography was used for nonpalpable undescended testes. Data were collected through completing the questionnaire by parents. 

The information including referral age, age of diagnosis, parent's awareness of surgery necessity, UDT and its associated complications, level of parents literacy, type of cryptorchidism (unilateral or bilateral) and place of living were recorded.

**Statistical analysis**

Statistical analysis was performed using the SPSS 13.0 software. Data are presented as frequency and percentage. Chi-Square test was used for data analyzing. Statistical significance was set at p < 0.05. 

## Results

In this study, the maximum recommended age of orchiopexy was 18 months. This is based on a summary of surgical, anesthetic, and psychological reasons (15). The mean age at referral was 5.34 years (range 20 days to 46 years) that only 44 (30.8%) cases were operated on before the age of 18 months. In patients which operated after 18 month, 17 (11.9%) cases were referred in 18-24 months, 48 (33.5%) cases in 2-7 years, 23 (16.1%) cases in 7-13 years and 11 (7.7%) cases were more than 13 years. Overall 99 (69.2%) patients were referred after 18 months. The most common reason of delay in surgery was the absence of early diagnose of cryptorchidism (42.5%). Other reasons were parent's unawareness of surgery necessity and UDT complications (33.7%) and disregard of them (23.5%). 28 (19.6%) of cases were diagnosed at born in the hospital. 31 (21.7%) cases were discovered by physician before 18 months as screening examination, while 19 (13.3%) of patients were diagnosed after 18 months. 65 (45.5%) cases were reported by parents. Seventy (49%) of parents had the correct information about the proper age for doing the operation, also 58 (40.6%) of them had enough information about necessity of surgery and side effects of disease (Table I). 

In evaluation of cause of delay, according to the literacy of parents, there was no significant differences (p>0.05). Also, type of cryptorchidism (unilateral or bilateral) and place of living (urban or rural) didn't have any significant relation with delay diagnosis (p>0.05) (Table II).

**Table I:** Parents awareness of proper operation age and necessity of surgery and side effects of cryptorchidism

	**Proper operation age**	**Necessity of surgery and side effects**
Correct information n (%)	70(49%)	58(40.6%)
Misinformation n (%)	16(11.2%)	11(7.7%)
No information n (%)	57(39.8%)	74(51.7%)

**Table II:** Cause of delay in proper treatment according to parents literacy, place of living and type of cryptorchidism

	**No early diagnosis**	**Parent's unawareness of surgery necessity and UDT complications**	**Parent's disregard**	**p-value**
Parent’s literacy, n (%) Mother Higher degree Lower degree Father Higher degree Lower degree	11(42.3%) 31(43.1%) 14(37.8%) 27(45%)	7(26.9%) 26(36.1%) 12(32.4%) 21(35%)	8(30.8%) 15(20.8%) 11(29.7%) 12(20%)	0.526 0.539
Place of living, n (%) Urban Rural	34(42.5%) 8(44.5%)	27(33.8%) 6 (33.3%)	19(23.8%) 4(22.2%)	0.986
Cryptorchidism type, n (%) Unilateral Bilateral	34(43%) 8(42.1%)	28 (35.4%) 5 (26.3%)	17(21.5%) 6(31.6%)	0.594

Higher degree: Post-Diploma, Lower degree: Pre-Diploma

## Discussion

Purpose of early diagnosis and selective therapy in cryptorchidism is to avoid irreversibility of severe histological alteration able to compromise gonadal, especially germinal function. Men with a previous history of cryptorchidism are still arriving at infertility clinics, and testicular cancers appear to be 5-10 times more common after cryptorchidism than in the rest of the population (16). On the other hand the frequency of cryptorchidism is still increasing in some countries (17). The result of our study showed that 69.2% of patients were operated after 18 months, as the maximum recommended age of orchiopexy. In addition, the average of the age during the approach has been 5.34 when different level of electron microscopic changes, failure of gonocyte transformation and testicular atrophy has been accrued for patients in this age (18, 19). Guven *et al* in evaluation of undescended testis in older boys found that 32% of orchiopexies were performed in boys with at least 4 years of age (20). Davey showed that of 468 orchiopexies, 266 (57%) cases were more than 5 years old (21). In a large series study by Cendron *et al* of 759 patients, 45% underwent surgery after the age of 4 years (22). In this study, late diagnosis was the major reason accounting for delay orchiopexy which only 41.3% of cases discovered before 18 months. Other possible reasons for delay were parent's unawareness of surgery necessity and UDT complications and parent’s disregards. On the other hands, parent's unawareness is one of the important reasons for delay in treatment. We found that only 44% of parents had correct information about proper operation age and 39.8% of them had no sufficient insight. 11.2% of parents had misinformation. In necessity of surgery and awareness of cryptorchidism side effects, 40.6% of parents had correct information; while 51.7% of them had no information. Misinformation observed in 7.7% of parents. To explain the probable risk factors, parent’s literacy, place of living and type of cryptorchidism were evaluated, and there was no significant differences related to these parameters. The results of this trial confirm conclusions of previous studies. Seddon *et al* indicated that missed diagnosis at birth and delay in referring for treatment by physicians appear to be major factors responsible for delay diagnosis and treatment. In this regard, they recommended an intensive education of both the public and medical profession (23). Sarmah showed that failure of medical screening is most common reason for late diagnosis of cryptorchidism. Also, he recommended that physicians and other health workers undertaking surveillance procedures should be adequately trained in the technique of examining testes of young babies and children and aware to refer boys over 6 months with undescended testis to surgeons (24). Moreover, Guven *et al *concluded many late orchiopexies can be prevented by primary care provider and parent education to encourage early referral (20). Also, Raghavendran *et al* revealed that physicians are the main responsible for the late presentation of the patients and indicated that careful physical examination and screening at birth by obstetrician, pediatrician and other practicing physicians is necessity (25). Overall, until now there have been plenty of studies performed about late diagnosis of cryptorchidism which indicates that delayed orchiopexy is still a universal problem (26-29). The superiority of our study was assessment of parents’ information about the UDT, treatment necessity and its complications. We also evaluated probable risk factor involved in delay management of cryptorchidism such as parent’s literacy, place of living and type of cryptorchidism. This study has some limitations, as well. Initially, we had no pre or post operative semen analysis in adult patients. Moreover, samples could not be taken from the testes for histopathological studies.

## Conclusion

This study reveals that the main reasons in delayed diagnosis and treatment of UDT are 1/physician failed to diagnose UDT at birth or even during follow-up periods and 2/lack of insight in relation to parents. Therefore, education of society especially parents and careful physical examination of the babies at birth and regular follow-up until 18 months can prevent the delay in diagnosis and treatment of UDT.
